# Feasibility of on/at Line Methods to Determine Boar Taint and Boar Taint Compounds: An Overview

**DOI:** 10.3390/ani10101886

**Published:** 2020-10-15

**Authors:** Maria Font-i-Furnols, Raúl Martín-Bernal, Marijke Aluwé, Michel Bonneau, John-Erik Haugen, Daniel Mörlein, Johanna Mörlein, Núria Panella-Riera, Martin Škrlep

**Affiliations:** 1 IRTA-Product Quality, Finca Camps i Armet, 17121 Monells, Spain; ulras.mar@gmail.com (R.M.-B.); nuria.panella@irta.cat (N.P.-R.); 2 ILVO-Flanders Research Institute for Agriculture, Fisheries and Food, Animal Sciences Unit, 9090 Melle, Belgium; Marijke.Aluwe@ilvo.vlaanderen.be; 3 IFIP, Domaine de la Motte au Vicomte, 35650 Le Rheu, France; michelbonneaupro@orange.fr; 4 NOFIMA-Norwegian Institute of Food, Fisheries and Aquaculture Research, P.O. Box 210, N-1431 Ås, Norway; John-Erik.Haugen@Nofima.no; 5 Department of Animal Sciences, University of Göttingen, Albrecht-Thaer-Weg 3, 37075 Göttingen, Germany; daniel.moerlein@uni-goettingen.de (D.M.); johanna.moerlein@uni-goettingen.de (J.M.); 6 KIS-Kmetijski inštitut Slovenije, Hacquetova ulica 17, SI-1000 Ljubljana, Slovenia; martin.skrlep@kis.si

**Keywords:** boar taint, human nose, LDTD-MS/MS, REIMS, Raman, biosensors, colorimetric method, SWOT analysis

## Abstract

**Simple Summary:**

Due to welfare issues, the physical castration of male pigs is decreasing, and the entire male pig production is increasing. Fattening entire male pigs requires control due to the possibility of accumulating off odour/flavour called boar taint, which is mainly due to two compounds - skatole and androstenone. If carcasses with boar taint reach the market, it can cause a negative consumer reaction which may have economic consequences for the whole meat chain. Thus, it is necessary to sort out carcasses at the slaughter line. Today, a sensory quality control (human nose method) is used in some slaughter plants for this purpose. Detection by physical or chemical methods is also envisaged. A colorimetric method to determine skatole has been used in Danish abattoirs for decades, but it is foreseen that it will soon be replaced by the laser diode thermal desorption ion source coupled with a mass spectrometry equipment that allows a fully automated classification based on skatole and androstenone levels at speed line, with a delay of less than 40 min. Other potential methods such as the electrochemical biosensors, rapid evaporative ionization mass spectroscopy and Raman spectroscopy, still need further development and validation for an application at abattoir level.

**Abstract:**

Classification of carcasses at the slaughter line allows an optimisation of its processing and differentiated payment to producers. Boar taint is a quality characteristic that is evaluated in some slaughter plants. This odour and flavour is mostly present in entire males and perceived generally by sensitive consumers as unpleasant. In the present work, the methodologies currently used in slaughter plants for boar taint classification (colorimetric method and sensory quality control-human nose) and the methodologies that have the potential to be implemented on/at the slaughter line (mass spectrometry, Raman and biosensors) have been summarized. Their main characteristics are presented and an analysis of strengths, weaknesses, opportunities and threats (SWOT) has been carried out. From this, we can conclude that, apart from human nose, the technology that arises as very promising and available on the market, and that will probably become a substitute for the colorimetric method, is the tandem between the laser diode thermal desorption ion source and the mass spectrometry (LDTD-MS/MS) with automation of the sampling and sample pre-treatment, because it is able to work at the slaughter line, is fast and robust, and measures both androstenone and skatole.

## 1. Introduction

Classification of pig carcasses and pork meat at the slaughter line allow both for an optimisation of its processing and for a transparent payment to producers. For these purposes, carcass grading is carried out in most countries using several types of devices, either manual, semi-automatic or fully automatic, based on a ruler, ultrasounds, reflectance, vision, electromagnetism or impedance [1,2,3]. In some countries, including those of the European Union, carcass grading is well defined by the legislation. The lean yield is the main information obtained, although its definition varies between countries (e.g., lean meat percentage, fat-free lean yield, saleable meat yield) and can be modified over the years. The lean yield, together with the carcass weight, can be used to establish the price of the carcasses [1,4] and to steer production in order to obtain carcasses with the desired characteristics. Some of these devices, as well as others used in the cutting plant, are mainly based on vision or X-ray. These systems also allow the classification of the carcass cuts and the optimization of the processing according to its characteristics [5].

Integrating on/at line determination of meat and fat quality characteristics in the classification of carcass (and cuts) would be a very powerful tool for meat industries to increase their market position. Several spectroscopic and imaging technologies have the potential to be used on line for meat quality determination [6,7,8]. Fat quality classification at the slaughter line is used in some countries like Switzerland, where the iodine and the PUFA (polyunsaturated fatty acids) values are analysed at line with a NIRS device and used to pay the producers according to national rules [9]. Seven slaughter plants (4 in Europe, 1 in US, 1 in Canada and 1 in South Korea) also use this parameter to classify carcasses by means of a portable device based on NIRS technology (NitFOM, Frontmatec A/S, Smørum, DK) [10]. At least two of them use it to pay producers (Mette Christensen, personal communication). They do so on their own initiative, not because of legislation requirements. Similarly, pH is measured on line in some abattoirs or cutting plants to classify pieces (e.g., ham) in order to optimize its further processing (e.g., cooking, drying and curing). To our knowledge, boar taint is the only other meat quality characteristic used for industrial quality control. It is measured at line in Danish abattoirs by means of a colorimetric technology, which determines skatole equivalents, or on line/at line in some Belgian, Danish, Dutch, French, German and Spanish slaughter plants by means of a sensory quality control evaluation (human nose). No legislation regarding this classification is available, although there is an EU Regulation [11] which states that meat with a pronounced sexual odour is unfit for human consumption. Moreover, in Germany there is legislation in place that describes both the sampling and the sample treatment for sensory evaluation to be carried out by veterinary inspection.

Boar taint is generally described as an unpleasant odour/flavour for sensitive consumers (e.g., Panella-Riera et al. [12]) that can be found mainly in meat from some entire male pigs and in a very low proportion in castrates and females [13]. The two main compounds responsible for boar taint are androstenone (5α-androst-16-ene-3-one) [14] and skatole (3-methylindole) [15,16] even though several other compounds have also been indicated to be involved, like androstenols [17], indols [18] and 4-phenyl-3-buten-2-one [19], as reviewed by Zamaratskaia and Squires [20]. Lately, 2-aminoacetophenone was also suggested to contribute to boar taint [21]. The detection of boar taint is important because boar taint can negatively affect consumers’ acceptability [12,22,23]. To avoid boar taint, surgical castration without anaesthesia/analgesia is widely used. Nonetheless, due to animal welfare issues, alternatives to surgical castration are also applied, including surgical castration with anaesthesia/analgesia, immunocastration or entire male production. In the latter case, breeding in addition to different feeding or management strategies is often used to reduce the incidence of boar taint. Between 5.5 and 56.0% of entire male carcasses exhibit high androstenone content (usually higher than 1 μg/g), 6.6 to 34.0% of them exhibit high skatole content (usually higher than 0.2 μg/g) [24,25] and 0.0 to 17.0% of them are detected as tainted by sensory assessment [26,27,28]. Because entire male pigs production is currently increasing [29], the development of methods for sorting out tainted carcasses on/at line is increasingly required. Classification according to boar taint as a meat quality parameter could be a meaningful tool to improve the present carcass classification systems and further support strategies for the reduction and masking of boar taint.

Over the years, numerous chemical methods have been developed to determine boar taint compounds mainly based on liquid or gas chromatography [18,30]. Whilst direct measurement with chemical methods can be robust, repeatable, reproducible and objective, these methods always require sampling and sample preparation, i.e., the selection of a representative sample, extraction and isolation of the target compounds from the matrix. Chemical methods are therefore time-consuming, cost-intensive and mainly restricted to the laboratory. However, some of these chemical methods have been adapted and automated to be used at line in the abattoir [31,32]. Systematic biases exist between the various available methods pointing out the need for harmonization [33,34]. To address this, the European Union established a reference method to determine the levels of androstenone and skatole [35,36].

Boar taint classification can be performed on line (measurements made directly on the carcass on the slaughter line) or at line (sample is removed from the stream and measurements are made in a separate room in the slaughter plant). It may be based on the determination of androstenone and skatole levels (whilst applying defined rejection thresholds), or on the sensory assessment of boar taint presence. This determination can be a direct measurement of androstenone, skatole or boar taint levels or an indirect measurement, i.e., a prediction of the compounds. Within the BoarCheck project [37] a deep analysis of rapid methods for boar taint determination used or being developed at that time was performed, but in recent years there have been improvements in some of them, and new methods have emerged. Within the Cost Action IPEMA (CA 15215), the Work Group 4 on Meat Quality was devoted to identifying recent innovation in grading and meat quality control systems. The aim of this paper is to review the methods that are already used or currently feasible for on/at line determination of boar taint in pig carcasses, to evaluate their characteristics and performance and to determine their strengths, weaknesses, opportunities and threats (SWOT analysis).

## 2. Description of on Line/at Line Available Methods

On line methods detect boar taint or boar taint compounds at the slaughter line level without taking samples from the carcass. They implicitly need to be rapid, can be connected to the slaughter line network to capture results at individual carcass level and are robust enough to be used in the working conditions of the abattoirs. At line methods may also be used at the abattoir although not just beside the line but in a separate room close to the line and thus require sampling. They are also rapid in the determination of boar taint or boar taint compounds, enabling a result to be produced before the carcasses leave the slaughter plant. The methods already used are the sensory evaluation by a trained panel (on line or at line), a colorimetric method (at line) and eventually a method based on LDTD-MS/MS (at line). Among the methods that show interesting results are the rapid evaporative ionization mass spectroscopy (REIMS), Raman spectroscopy and electrochemical biosensors. These methods are presented in the next sections of this paper. There are other potential methods in development such as gas phase measurement combining Fourier Transform Infrared and Photo Acoustic Spectroscopy, gas phase fingerprint and gas-sensor array, immunological methods, insect based biosensors [37] and solid phase microextraction coupled to gas chromatography/mass spectrometry [38], but they still need further development and validation to be suitable for its portable version to be used on line, to increase their capacity, automation or to improve performance.

All the methods described here perform the analysis of boar taint, androstenone or skatole in fat instead of meat tissue. Androstenone and skatole are accumulated in fat [39] which is a good indicator of the level of boar taint, although skatole and androstenone are lower in meat than in fat [40,41,42].

### 2.1. Sensory Evaluation (also Referred to as Human Nose Scoring)

#### 2.1.1. General Aspects

Sensory evaluation allows a quantitative and qualitative characterization of foods. Analytical sensory methods are often used by food industries for research and development, and as a quality control tool in various industries. There are official sensory quality protocols available (i.e., for virgin olive oil [43]). Analytical sensory assessments are carried out by trained panellists/assessors and their evaluation can impact the industries’ benefits [44]. Inter- and intra-individual variability in olfactory (and other sensory performances) is inherent to humans. It can be controlled by using a group of assessors instead of a single assessor. As for any other chemical/physical method, quality control specifications need to be defined in order to identify and control the variability of the assessors. An objective recruitment and training of the assessors and regular evaluation and documentation of their performance are therefore critical to identify suitable assessors and to have an accurate, efficient and reliable panel [45]. Refreshment and regular training (i.e., re-calibration) are necessary to ensure an accurate and consistent assessment over time. Moreover, at industrial level, it is important that plant managers maintain the motivation of the assessors, and that they respect and support them, to avoid negative effects on the panel performance [46].

#### 2.1.2. Boar Taint Evaluation

**Figure 1:**
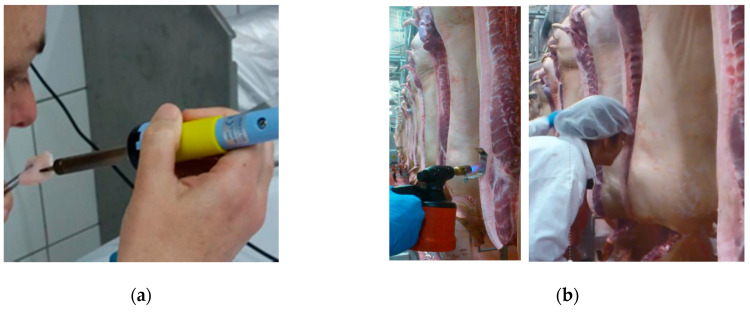
Human nose performed (**a**) at line and, (**b**) on line at a slaughter plant.

A sensory quality control of boar taint performed by sensitive and trained assessors (classifiers) on/at line is known as human nose methodology [26]. The method classifies carcasses usually as tainted and non-tainted or in some cases using a scale according to different degrees of taint [26,47]. It can be used either at-line when samples are taken out for assessment in a laboratory (Figure 1a) or directly on-line where (most often) fat tissue is heated directly (Figure 1b).

The following steps are needed to carry out the sensory evaluation:

##### Selection and Training of Assessors

Assessors must be selected according to their ability to perceive both key boar taint components given the above described inter-individual variability in olfactory acuity to both androstenone and skatole and the well-known anosmia to androstenone that strongly influence the individual perception and thus sensory evaluation results [47,48]. For selection of assessors, thresholds for assessors’ sensitivity need to be defined in line with the specific requirements of the company. For example, a limit of detection for boar taint compounds in reference or native back fat samples could be specified; this should be aligned with the sorting limits of that given company. An underlying assumption is that, if highly-sensitive and well-trained assessors cannot detect boar taint under well-controlled conditions, it becomes unlikely that a naive consumer will detect it when eating pork at home, i.e., in a less controlled and more variable environment. It can, however, not be ruled out that some consumers are even more sensitive than the assessors.

**Figure 2:**
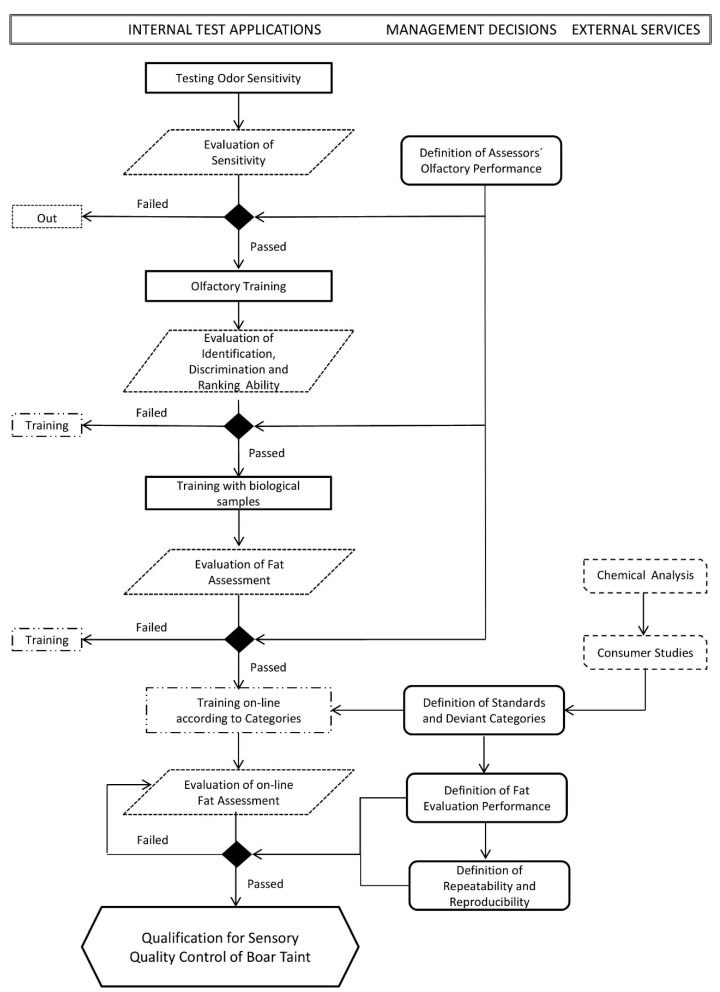
Flow chart of assessor qualification for sensory quality control of boar taint (adapted from [45]).

Training of the assessors consists of the following steps represented graphically in Figure 2:

Step 1: Olfactory training: 

Specific olfactory training with boar taint compounds must be the starting point in assessor training. Discrimination tests should be performed to objectively evaluate the assessors’ ability to correctly discriminate androstenone and skatole from each other. Further training in identification and ranking is necessary. The training can be done using chemical substances such as 5α-androst-16-en-3-one and 3-methylindole, either mixed with oil [48,49], in bottles with liquid [26] or spread/dried on smell strips [50].

Step 2: Training with biological samples (i.e., fat samples) (Figure 1a). 

Training should also be carried out using native fat samples, with known levels of androstenone and skatole [26,47,48]. As an example, samples used by Trautmann et al. [26,47,48] presented androstenone and skatole levels up to 4.0 and 0.41 μg/g, respectively and the thresholds used for classification as tainted established at 1.5 μg/g and 0.2 μg/g for androstenone and skatole, respectively. However, levels of biological samples will depend on the selection performed at slaughter plant and availability of tainted carcasses. Training can also be performed with fat samples spiked with known concentrations of synthetic androstenone and skatole [51]. This is however not recommended as spiked samples do not smell like samples with naturally occurring boar taint compounds (Lunde, personal communication). The performance of individual assessors and the panel (i.e., group of assessors) can be determined by calculating their sensitivity, specificity and accuracy, as well as repeatability and reproducibility [26,47,52]. Both chemical measurements and/or the average panel evaluation can be used as reference for its determination. All these performance criteria help to get to know the panel and, consequently, to decide the minimum number of assessors required to achieve the desired accuracy or the target cut-off criteria of the industry [26].

Step 3: Training on line (Figure 1b). 

If the assessors are going to classify on line, i.e., directly on the carcass, a further step should be performed in order to train assessors to perform the evaluation in working conditions. In this case, parallel testing by more than one assessor is needed, followed by discussion to reach an agreement and to establish the scale of measure [26].

##### Sample Preparation

Due to the chemical properties of boar taint compounds (low volatility), perception of androstenone and skatole is facilitated by heating the sample. This can be done using different technologies, such as microwaves, soldering iron, gas powered torch heated plate or hot water. The gas powered torch heated plate is the most commonly used for the on line method. Trautmann et al. [53] compared three sensory protocols for boar taint determination at line with 10 trained assessors, using microwaves, hot-water or hot-iron and concluded that hot-iron was the best in terms of sensitivity and specificity validated against a chemical method. In another work, Whittington et al. [54] compared microwave, hot wire, melting and water (at 75 and 25 °C) using three trained assessors and concluded that microwave, hot wire and water at 75 °C were the best to find out differences in odour between samples. Bekaert et al. [55] compared microwave, pyropen, pyropen with plate and soldering iron, showing higher boar taint scores for microwave and soldering iron devices. Usage of the same heating method during training as the one that will be eventually be used is highly recommended.

##### Performance of the Analysis

As for any other quality control tool, the performance of a sensory evaluation method must be objectively evaluated using statistical parameters such as sensitivity and specificity. It is important to note that the results of such a performance evaluation depend on the assessors and on what is considered as the “gold standard” reference (i.e., a standardized chemical method). The reference that defines the true value can differ between studies making comparisons more difficult (see Section 3.5). Using 10 assessors, Mathur et al. [26] reported a sensitivity between 0.08 and 0.29 and a specificity between 0.91 and 0.99 when androstenone and skatole levels were used as gold standard (thresholds for tainted carcasses were 1 μg/g for androstenone and 0.25 μg/g for skatole measured in liquid fat). In the same study, performance ranged from 0.61 to 0.82 for sensitivity (average 0.75) and from 0.85 to 0.98 for specificity (average 0.94) when, instead, the average of the human nose score was used as gold standard (tainted if average score of panellists ≥ 2.5 in a scale from 0 to 4). Reproducibility varied between 0.18 and 0.32. When thresholds for chemical values were adjusted to higher values, sensitivity became comparable between both reference values used. Trautmann et al. [53] using 10 assessors, reported a sensitivity between 0.68 and 0.88 and a specificity between 0.68 and 0.91 depending on the heating method (microwave, hot-water and hot-iron) when tainted carcasses were considered those with ≥1.5 μg/g of androstenone or ≥0.20 μg/g of skatole in melted back fat. When the (chemical) thresholds for classification as tainted were higher (≥2.0 μg/g of androstenone or ≥0.25 μg/g of skatole in melted back fat), the sensitivity varied between 0.72–1.0 and specificity between 0.63–0.85, depending on the heating methods. Accuracy varied between 0.46 and 0.78 (average 0.61). Using 18 assessors (5 evaluating the same sample), Meier-Dinkel et al. [56] found an average sensitivity of 0.82 and specificity of 0.67 when using the average panel score as reference (cut-off threshold ≥ 2 in a scale from 0 to 5). These averages were 0.68 and 0.63 for sensitivity and specificity, respectively, when the gold standard was the chemical determination of androstenone and skatole with cut-off thresholds for tainted carcasses with ≥1.5 μg/g of androstenone or ≥0.20 μg/g of skatole in melted back fat and it were 0.74 and 0.61, respectively when cut-off thresholds were ≥2.0 μg/g of androstenone or ≥0.25 μg/g of skatole in melted back fat.

When sensory evaluation is carried out on line, van Wagenberg [56] established that one assessor could smell for a maximum of 30 min to avoid fatigue, needing 15 min of rest between evaluations. This work established a maximum of 1000 evaluations per day.

To improve the performance, it is important to clean the tip of the soldering iron after each sample to avoid cross-contamination, smell a blank sample after a tainted sample, carefully monitor the heating temperature (if too high, a burning smell will cover everything) and alternate between assessors to avoid fatigue and saturation [55]. Performance will also improve if more than one assessor evaluates the same carcasses, as detailed by Mathur et al. [26]. If the line speed is too quick, more than one assessor is needed in order to evaluate alternate carcasses.

##### Implementation Costs

The cost of this methodology is mainly related to human resources because the evaluation is usually performed by personnel of the abattoir. The cost of the evaluation of a sample would mainly be the hourly salary of the operator or the operators (if more than one assessor evaluates the same carcasses to improve performance) to carry out the analysis divided by the number of carcasses evaluated each hour. Additional costs need to be considered such as time spent in training sessions, trainer salary and material for training. Further costs include the depreciation of the initial investment, e.g., when a sensory lab is used or for equipment to record the scores. Also, because of the necessity to alternate between assessors to avoid saturation or due to high speed of the slaughter line, more than one assessor is required to perform the evaluation. 

### 2.2. Colorimetric Method

#### 2.2.1. General Aspects and Boar Taint Determination

A fully automated colorimetric method was proposed by Mortensen [57]. The method is based on the reaction between a colour reagent and skatole and other indolic compounds, and has been used at line in the slaughterhouses of Denmark since the 1990s. Due to the colour reagent interacting with, and only with, all indolic compounds, the method is not fully specific for skatole and does not measure androstenone.

#### 2.2.2. Sample Preparation and Analysis

A fat sample is excised from the carcass. Four grams of fat are minced and mixed with 40 mL of extraction solution [58]. The colour reagent used was 4-dimethylaminobenzaldehyde and was added to the sample mixture for 3 to 5 min before reading the absorption at 580 nm on a spectrophotometer. This process was further automatized using the Technicon II system. The results are expressed as skatole equivalents per g fat tissue (μg/g).

#### 2.2.3. Performance of the Analysis and Speed Capacity

The response time (from sampling to result) is 10 to 20 min and up to 360 samples per hour can be analysed [59]. Accuracy of the method was determined by performing the measurement in duplicate on a total of 120 samples. This resulted in a correlation coefficient of r = 0.94 between repeated measurements. Recovery of the skatole added into the samples before starting the extraction process was between 95 and 105% while the limit of detection ranged from 0.02 to 0.04 μg/g skatole equivalent units [58].

#### 2.2.4. Implementation Costs

The cost of this methodology includes the depreciation of the initial investment of the sampling device, the sample conveying systems, the room to perform the analysis, the Technicon system and a spectrophotometer. Running costs include the reagents needed to carry out the analysis, the salary of the technician/s to take the samples, perform the analysis and interpret the results, maintenance of the device, and disposal of the residues generated in the analysis.

### 2.3. Laser Diode Thermal Desorption Ion Source Tandem Mass Spectrometry

#### 2.3.1. General Aspects

**Figure 3:**
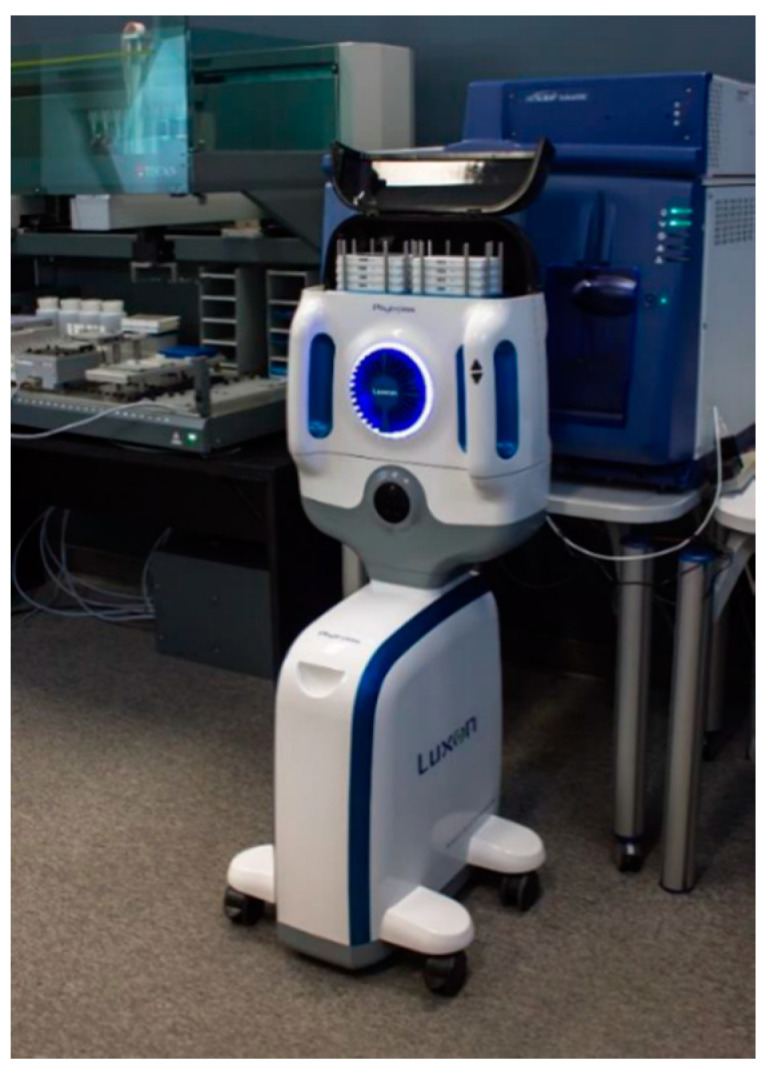
Laser diode thermal desorption ion source tandem mass spectrometry (LDTD-MS/MS) analysis (Source: [61]).

New devices based on a laser diode thermal desorption ion source tandem mass spectrometry (LDTD-MS/MS) have been presented separately by the Danish Technological Institute (DTI) (Figure 3) and Shimadzu Corporation as an option to determine the presence of tainted carcasses at slaughter [31,60]. This technology enables the injection of the analytes in gas phase directly into the ion source of the mass spectrometer, without any prior chromatographic separation, which is time consuming.

#### 2.3.2. Sample Preparation and Analysis

Borggaard et al. [31] established a procedure to sample 0.3 to 0.8 g of back fat, place it into a 24 deep well plate (DWP), transfer the DWP to the LDTD-MS/MS device and add 1.5 mL of brine and 1.5 mL of acetonitrile containing internal standards (5α-Androstan-3-one and deuterated skatole). After homogenization for 30 s, centrifugation is performed (5000× *g*, 5 min) and 4 µL of supernatant is transferred to LazWell^TM^ microtiter plates for desorbing, ionizing and measuring the analytes with two-dimensional mass spectrometry (MS/MS).

Auger et al. [60] described a simpler means of sample preparation that needs further validation: 0.3 g of back fat is weighted in a tube together with 3 mL of NaOH (1 N in water) and 3 mL of internal standards (Androstenone-d_4_ and Skatole-d_3_). The mixture is vortexed until fat dissolution (3000 rpm, 1 min) and left to rest until phase separation (2 min); 4 µL of supernatant, upper layer, are placed in a LazWell™ plate and there is a solvent evaporation (1 min at room temperature).

LDTD-MS/MS analysis continues as follows: after sample pre-treatment and a small amount of sample placed on a well plate and once the solvent evaporation is done or nearly done, a laser diode heats the back of the well plate transferring heat to the dried sample. The analyte is vaporized and carried along, together with the carrier gas, through the transfer tube until the corona discharge, where the analyte is chemically ionized at atmospheric pressure (APCI).

#### 2.3.3. Performance of Analysis and Speed Capacity

In the WIPO patent, WO2016139291 Lund and Borggaard [62] described that less than 10 s of time is needed between samples, leading to 360 samples analysed per hour. Borggaard et al. [31] reported a limit of quantification of 0.1 µg/g of fat for androstenone and 0.05 µg/g of fat for skatole and a limit of detection of 0.05 and 0.02 µg/g of fat for androstenone and skatole, respectively.

#### 2.3.4. Implementation Costs

The cost of this methodology includes the depreciation of the initial investment for the acquisition of the mass spectrometer, the laser diode thermal desorption ion source and the surrounding lab environment. Running costs include the reagents needed to carry out the analysis, the salary of the technician taking the sample, performing the analysis and interpreting the results, the maintenance of the device, and a cost for disposal of the residues generated in the analysis. Additional depreciation and maintenance costs will emerge if sampling and sample pre-treatment are automated to facilitate the use of this technology at abattoir level, but some personnel costs can then be avoided.

### 2.4. Rapid Evaporative Ionization Mass Spectroscopy

Rapid evaporative ionization mass spectroscopy (REIMS) was first proposed by Schäfer et al. [63] for a medical application. It is based on the rapid thermal evaporation of tissue constituents by reaching molecular evaporation rates comparable to the rate of decomposition, which results in the formation of gaseous molecules or molecular ions. The ion source used is an electrosurgical electrode, coupled with a mass spectrometer. The electrosurgical electrode evaporates the tissue sample and both positive and negative ions generated are transferred to the mass spectrometer using a Venturi pump.

Verplanken et al. [64] applied this technique for boar taint determination. They used the iKnife hand-held sampling device (Waters, Wilmslow, UK) as an electrosurgical electrode coupled with a TOF-MS, with a lecture time of 3–5 s/sample and 2 technical replicates. The iKnife, transfer tubing and Venturi pump were cleaned with methanol after 10 samples. The technique does not measure androstenone and skatole levels. A mass spectrum is taken of the volatile compounds generated by the application of the iKnife on the adipose tissue surface. Chemometrics are used to build discriminant models to classify sow samples, non-tainted boar samples and tainted boar samples according to its boar taint or skatole/androstenone levels. This discriminant analysis seems to be mainly based on mass spectra of the fatty acids from the major lipids, i.e., glycerides and phospholipids, as shown by spectral differences in negative ionization mode between classification groups.

#### 2.4.1. Performance Analysis

A discrimination model, using orthogonal partial least-square discriminant analysis (OPLS-DA), was built with both positive (50) and negative (50) boar taint carcasses (positive tainted if indole, skatole and/or androstenone were above 0.1, 0.2 and 0.5 μg/g, respectively) as well as sows (50) in order to obtain a fingerprint of the boar taint mass spectra and construct predictive models. After elimination of 5 outliers, all blank and negative samples were correctly classified by cross validation whilst they achieved 98% accuracy for tainted carcasses (OPLS-DA model), taking chemical (androstenone and skatole levels measured by UHPLC-HRMS) and sensory evaluation as a reference. Robustness was tested by reducing the heater power of the settings which resulted in a lower accuracy (89%) for the correct classification of the three groups of samples. Recently, REIMS has been tested at slaughter plant level, sampling carcasses every 10 s. For this purpose, a calibration model was previously developed in a laboratory using 1097 samples and, later, the training phase was repeated in situ at the abattoir in 554 samples and classified tainted carcasses using an untargeted AMS (ambient mass spectra) approach, but no performance data has been provided [32]. Indirect techniques based on “profiles”/fingerprinting have proved in the past to be insufficiently robust to take into account the variability of situations occurring in commercial slaughterhouses with pigs from different genotypes coming from different farms and fed diets with different lipid composition [65], so further information and validation is needed.

#### 2.4.2. Implementation Costs

The cost of this methodology includes the depreciation of the initial investment for the acquisition of the mass spectrometry iKnife hand-held sampling device. It is also necessary to include as running costs the reagents needed to carry out the analysis, the salary of the technician to perform the analysis and interpret the results, and the maintenance of the device. There is also a cost to move away the residues generated in the analysis.

### 2.5. Raman Spectroscopy

#### 2.5.1. General Aspects and Applications

Raman spectroscopy is a high-resolution photonic technique that provides both qualitative and quantitative information on the physico-chemical nature of a material. Described by the Indian physicists Raman and Krishnan [66], it is based on the irradiation of the sample with monochromatic light and the later analysis of the information contained in the inelastic scattered light. The Raman effect is based on the interaction between the photons and the vibrational and rotational movements of the molecules of a given sample. This is the result of the interaction between the photons and the vibrational and rotational movements of the bond molecules of the sample. Briefly, the incident photon leads transiently the molecule to a higher vibrational (or rotational) energy level, which it quickly abandons to pass to a more stable energy level emitting a photon with a new frequency [67]. The difference in frequency between the incident and scattered radiation is known as Raman shift.

Raman spectroscopy has a huge potential and, due to its portability and general performance, it has been used in pollutant environmental detection [68], pigment identification on painted artwork [69], raw material identification, product identification, and quality control in the pharmaceutical industry [70], detection of biomarkers for biomedical science [71,72], as well as in a wide range of situations in the food industry [73].

#### 2.5.2. Boar Taint Evaluation

Given its speed, non-invasiveness and simple use, Raman spectroscopy has been proposed as a candidate option for the determination of the boar taint compounds, androstenone and skatole in pork fat [74,75]. The method does not directly measure androstenone and skatole. Chemometrics are used to build a partial least-square discriminant analysis (PLS-DA) model to predict the boar taint compounds. The Raman spectrum seems to indicate that the discrimination between groups of boar taint is based on the fatty acid profile which has some statistical relationship with the levels of boar taint compounds [75]. This indirect relationship implies a weakness as a calibration model would need to cover the well-known variability of fatty acid composition of the tissue lipids according to genotype, sex and fatty acid composition of the diet.

##### Sample Preparation

Raman technique can be applied directly on the fat surface, facilitating its use on/at line. Wang [74] used a total of 105 pork fat samples with known levels of androstenone (<0.02 to >2 µg/g) and skatole (~0.03 to 0.7 µg/g). Sample preparation only required removing muscle tissue and mounting the clean sample onto microscope slides for measurement.

Liu et al. [76] worked with a total of 46 back fat samples with androstenone levels ranging between <0.5 to ~7 µg/g and skatole levels from <0.2 to ~1.5 µg/g (all values reported for melted fat). Due to congelation conditions during the storage, samples were defrosted at 4 °C for about 16 h, followed by an acclimation to room temperature (18–22 °C) of 1 to 1.5 h. In this case, the authors separated the fat layers (outer and inner) to determine whether there were differences between them in the data acquisition.

##### Performance of Analysis and Speed Capacity

Wang [74] performed the analysis with a DXR Dispersive Raman Microscope (Thermo Scientific, Inc., Madison, WI, USA), with 780 nm of wavelength. Conditions for data acquisition were 14 mW for the excitation laser, 2 s of exposure time and a range between 200 and 2800 cm^−1^ with a resolution of 1 cm^−1^; they acquired at least 5 replications per sample, all of them at room temperature. The best value of accuracy reached in the publication showed ~92% for high concentrations of androstenone and skatole.

The device used by Liu et al. [76] was a hand-held Raman sensor [77] with an excitation wavelength of 671 nm. The spectra were collected in the ranges of 300 to 2100 cm^−1^, with 6 s of integration time, 10 accumulations per spectrum and a laser power of 50 mW. Per sample, 20 replicates (10 per fat layer) were carried out. Altogether, total acquisition time took 20 min per sample; from this, an analysis time of about one minute per sample can be determined. Finally, after removing outliers and using a specific range of measurement (958–972 cm^−1^ and 1354–1370 cm^−1^), they showed an accuracy of 81%, a sensitivity of 72% and a specificity and 88% whereby the reference (gold standard) was a classification into tainted/untainted samples based on sensory evaluation (tainted if average panel score ≥2, in a 6 point scale from 0 to 5) as well as chemical analysis of androstenone and skatole (thresholds applied were 1.5 μg/g for androstenone and 0.2 μg/g for skatole).

Indirect assessment of skatole and androstenone levels via a discriminant model, probably linked to fatty acid composition of the tissue, is unlikely to be accurate in commercial slaughterhouse conditions where pigs from different genotypes are coming from different farms and receiving diets differing in their fatty acid composition.

Based on Raman spectroscopy, Sørensen et al. [78] used a surface-enhanced Raman scattering (SERS). This modification of the Raman technique is carried out in an improved environment (nanoparticles, a determined pH and aggregating agents) that allows an indirect quantification using multivariate techniques such as partial least-square (PLS) and interval PS (iPLS). Comparatively to the regular Raman spectroscopy, SERS has the advantage of measuring androstenone and skatole levels. However, in the present stage of development of the technique, the observed prediction errors are much too high (0.17 μg/g for skatole, 1.46 μg/g for androstenone) [78] to make the method of any use in boar taint detection.

##### Implementation Costs

The cost of this methodology includes the depreciation of the initial investment for the acquisition of the Raman fix device or the Raman hand-held device. It is also necessary to include the running costs of the technician to take the sample if the measure is at line, the technician to perform the analysis and interpret the results (unless a built-in model does the data analysis and subsequent classification as is done typically in industrial hand-held devices) and the maintenance of the device.

### 2.6. Electrochemical Biosensors

#### 2.6.1. General Aspects and Applications

Biosensors based on screen-printed carbon electrodes are electroanalytical devices that allow the detection and quantification of a target compound via the oscillations of differential pulse voltammetry (DPV) provoked by the activity of a specific enzyme in presence of the before mentioned target molecule. Basically, the working electrode is made of carbon ink together with the enzyme immobilized onto its surface and a mediator. The mediator actively uses the nicotinamide adenine dinucleotide (NAD) electrons to be reduced and then oxidised, which constitutes the analytical signal. The crux of the matter is that the target compound is acting as a cofactor for the enzyme used; in presence of the molecule (androstenone and/or skatole), the enzyme becomes reduced and the molecule oxidized due to the cofactor NAD, which is acting as an electron carrier. As long as the enzyme is competing with the mediator, in presence of the target compound, the lecture of the signal will be altered proportional to the quantity of the monitored molecule (e.g., [79]).

Biosensors can be applied at a wide spectrum of different fields: for the detection of drug molecules like benzodiazepines [80], vitamins (e.g., [81]), environmental pollutants [82], molecular biology [83], and even for the boar taint compounds androstenone and skatole [84].

#### 2.6.2. Boar Taint Determination

**Figure 4:**
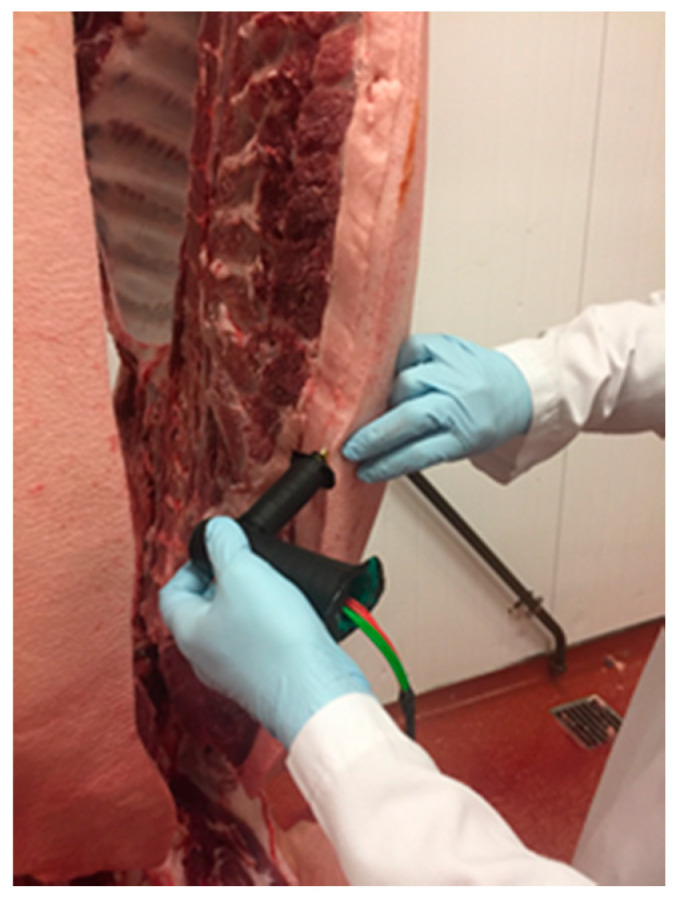
Evaluation of a carcass with University of West England (UWE) Electrochemical biosensor system (photo courtesy of O. Doran, A. Crew and J. Hart).

Westmacott et al. [84] designed a portable device for the measurement of androstenone and skatole levels at the slaughter line. This is composed by a potentiostat (µAutolab III, Metrohm, Netherlands) connected to a PC for data acquisition, a working electrode for the detection of skatole made of carbon ink (C2030519P4) and a working electrode for the androstenone made of carbon ink modified with Meldola’s blue (BE2031028D1/24) and the enzyme 3α-hydroxysteroid dehydrogenase (3α-HSD). On line measurement can be performed via automated static or manual portable devices for in situ use (O. Doran, A. Drew and J. Hart, personal communication). Figure 4 shows the use of a biosensor directly on the carcass.

Biosensors can target from single atoms to complex proteins and, depending on the ligand, the desirable assay time could vary, as could the sensitivity, the lower and upper range of measurement or the stability of the biosensor [79]. All these characteristics could be modified according to the immobilization technique and the mediator used. In the case of the androstenone biosensor, Meldola’s blue is used as a mediator and the enzyme 3α-HSD is immobilized by drop coating onto the electrode surface.

According to the authors, both sensors are disposable avoiding the cross contamination between samples. However, in large slaughterhouses (500–600 carcasses/h) the number of biosensors required as well as the time needed to replace them (up time) must be considered.

##### Sample Preparation

Hart et al. [85] reported that there is no need for sample preparation or sample pre-treatment. For the validation of the methodology, Westmacott et al. [84] established that an incision was required in the outer subcutaneous back fat layer in order to insert the sensor for the measurement; in the same study, calibration of the device was made only with the outer fat layer. If the at line scenario is considered, half carcases do not require an incision, but still the operator should perform the measurement in the outer fat layer. This situation could require further modifications of the calibration method if, for instance, the characteristics of the animals makes the outer fat layer thinner.

##### Performance of Analysis

The performance of a biosensor can be considered as optimal when linearity is observed between the amount of ligand and the obtained signal. Westmacott et al. [84] found this relation for androstenone between 0.3 μg/g–4 μg/g and for skatole between 0.052 μg/g–0.34 μg/g, leading its respective limits of detection in 0.3 μg/g for androstenone and 0.052 μg/g for skatole in pure fat. The correlations (coefficient of determination) observed between the gas chromatographic measurement and biosensors measurement in fat samples resulted in r^2^ = 0.93 for androstenone and r^2^ = 0.80 for skatole but it should be validated with higher number of samples. However, the accuracy of this method compared to the reference method was not very high. The recovery of the biosensors against as chromatography resulted in 95.9% (AND) and 114.5% (SKA). For both coefficient of determination and recovery, a total of 21 and 14 samples were used for androstenone and skatole analyses, respectively.

Measurement time reported in Haugen et al. [37] was 30 s and the total analysis time less than a minute. Westmacott et al. [84] determined a 10 s of deposition time and 2 min of response monitoring for androstenone determination. Since several biosensors can be used simultaneously, the run time is not highly relevant. However, biosensors need to be adapted to be used on line in slaughter plants [84]; thus, performance of the method in industrial conditions remains to be determined.

##### Implementation Costs

The cost of this methodology includes the acquisition of biosensors. It is also necessary to include the technician that will insert and extract the biosensor and do the analysis. If biosensors are reusable, the cost of cleaning them to avoid cross-contamination would need to be considered and a renovation plan is also needed when they need to be changed. To avoid cross-contamination, biosensors can be developed as disposable. In this case, they cannot be reused and one biosensor per carcass to be evaluated is needed and considered in the cost.

## 3. General Discussion and SWOT Analysis

The reviewed methodologies and technologies to determine boar taint or boar taint compounds, have some characteristics that are common between them and others that make them unique. When evaluating the methodologies for carcass and meat grading, aspects such as speed and capacity, invasiveness-destructiveness, automation, on line evaluation, performance, cost-effectiveness, multi-uses and correlation with consumers’ perception have to be considered [86]. In any case, and considering all these factors, the technologies/methodologies reviewed have strengths, weaknesses, opportunities and threats. The analysis of these characteristics (SWOT analysis) is presented in Table 1.

### 3.1. Technology Readiness Level

Technology Readiness Level (TRL) is a commonly-used scale that measures the maturity of technologies that stablish 9 levels [89]: TRL-1 (basic principles observed), TRL-2 (technology concept formulated), TRL-3 (experimental proof of concept), TRL-4 (technology validated in lab), TRL-5 (technology validated in relevant environment), TRL-6 (technology demonstrated in relevant environment), TRL-7 (system prototype demonstration in operational environment), TRL-8 (system complete and qualified) and TRL-9 (actual system proven in operational environment). Sensory (human nose scoring) and colorimetric methods have the strength of being already in use at abattoir scale to sort out tainted carcasses, so they are at Technology Readiness Level (TRL-9). Sensory evaluation by trained assessors allows a classification of carcasses according to their boar taint level directly on line (so no sampling is required). Despite the fact that usually sensory evaluation is done in dedicated labs the noisy environment of the abattoir does not affect their performance [90]. The colorimetric technique that quantifies skatole equivalents is used to sort out carcasses with high levels of skatole (above 0.25 µg/g). It is expected that soon this colorimetric method will be replaced by LDTD/MS-MS having the benefit that androstenone and skatole are quantified [58]. LDTD/MS-MS is complete and qualified, and as per its developers (DTI) is going to be proven soon under operational environment; thus, it is between TRL-8 and TRL-9. REIMS technology has been evaluated at line in abattoirs [32], but there is no evidence yet that the system works in different slaughterhouses with different samples, thus its readiness is considered to be TRL-5. Regarding Raman, although some of the works have been carried out with a portable device [75] it has only been validated in the lab, so it could be placed at TRL-4. Electrochemical biosensors have been evaluated directly on carcasses [84] and also a demonstration with a prototype in the abattoir has been carried out (O. Doran, A. Drew and J. Hart, personal communication). However, as with REIMS, there is no evidence yet that the system works in different slaughterhouses with different samples; thus, its readiness would be TRL-6.

### 3.2. Type of Measurement

Sensory evaluation via human nose assessment is used to classify carcasses according to its **direct assessment of off-flavour generally linked to boar taint**. This analysis is directly related to the sensitivity of the assessor or the panel of assessors, but probably also reflects the perception of consumers. Therefore, it might be preferred to chemical analysis as the gold standard. Hence, this is considered the strength of sensory evaluation, even though the performance should be evaluated carefully because the perception varies across humans. On the other side, LDTD/MS-MS technology allows a **direct measurement of androstenone and skatole**, i.e., the main compounds responsible for boar taint; it then is possible to classify carcasses according to their concentrations. Some works use a fixed threshold for each compound to classify tainted carcasses [91,92]. In 2000, Bonneau et al. [93] modelled the percentage of dissatisfied consumers depending on the levels of androstenone and skatole. Aluwé et al. [88] built a dissatisfaction map which provides the percentage of dissatisfied consumers, depending on the combination of androstenone and skatole of the meat, both considering the overall populations (given that 34% of consumers were tested sensitive to androstenone) and considering only the consumers sensitive to androstenone. In a recent study, Christensen et al. [87] built a risk-of-dislike model based on the consumer ratings depending on the levels of androstenone and skatole, and also considering the skatole and androstenone concentrations in the tested pig population. If the levels of androstenone and skatole are provided, the industry will need to decide the threshold according to the risk they are able to assume.

The colorimetric method is a long-established method successfully used to sort out boar tainted carcasses based on a **direct measurement of skatole** (in fact, skatole equivalents). This can be considered a weakness of the method because androstenone is not measured. However, although boar taint is related to both skatole and androstenone, there is a significant correlation between skatole equivalents and sensory evaluation by trained assessors [59], and also, some studies showed that skatole is the most important compound affecting consumers’ acceptability [93,94]. This is probably related to the fact that skatole is perceived by a higher number of consumers (from 59.6% to 99.0% [50,95]) than androstenone, which represented a higher proportion of anosmic consumers (from 17.5% to 73.0%; reviewed in [22]). Also, skatole is perceived at lower concentrations than androstenone [47,48] and is volatilized at lower temperature than skatole [96,97]. Nevertheless, the single determination of skatole is a weakness because for some consumers, especially those sensitive to androstenone, both compounds affect their pork acceptability [98].

Other technologies such as Raman, REIMS, SERS and biosensors perform an **indirect measurement** of boar taint compounds. From these, Raman and REIMS are classification techniques based on discriminant models, whilst SERS is a quantification technique based on multivariate calibration. This can be considered a weakness because the measure depends on other intermediate factors or compounds such as the composition of the fatty acids, mass spectrometric fingerprint, or the specificity of the enzyme for the target compounds. Moreover, in Raman and REIMS, the measurement is a prediction; thus, the error of prediction needs to be added to the error of detection. Also, the calibration models depend on the pig population used, results of this methodology might be affected by a high variability of type of animals that could be found among farms. It is therefore expected that it could be difficult to obtain sufficient robustness for use at abattoir level.

### 3.3. Traceability and Sample Pre-Treatment

Keeping **traceability** is crucial to ensure the good functioning of the methodology in order to sort out carcasses. Thus, a system that links the measurement of the result to the carcass identification number is required. In methodologies where the measure and the results are taken directly on the carcass and obtained **on line** (i.e., on line human nose) this could be easier because sampling is avoided and results are obtained immediately, reducing the possibility to lose track, which can be considered as a strength. It can be a weakness in methodologies where **sampling** is necessary, and the measurements are carried out at line (i.e., at line human nose, colorimetry and LDTD-MS/MS). Nevertheless, in all the cases, a good identification of the sample is a requirement throughout the whole process, and a sampling plan is needed to keep traceability. Methods that avoid sampling also have the advantage of being **non-destructive**, with the exception of methods such as human nose, which require heating (although only minimal damage is observed on the carcass). When sampling is required, it can be considered a destructive method, but the effect on the carcass is minimal because the amount of fat required for the analysis is very limited (i.e., 0.3–0.8 g fat in LDTD-MS/MS, [31]).

The lack of **sample preparation** is considered a strength of human nose, REIMS, Raman and biosensors methodologies and it could be a weakness in the other methodologies due to the increment of time needed to obtain results and this could complicate the possibility to adjust the method to be carried out on line. Nevertheless, if at the slaughterhouse carcasses are sorted out before the chilling room and results are available at that time (like with colorimetric methods and, potentially, also with LDTD-MS/MS), the sample pre-treatment does not delay the classification. Also, automated sampling and sample preparation can help to reduce the time to have the results of the analysis. In this sense, the Danish Technological Institute offers a fully automatic system for both sampling and sample preparation, as well as for analysis [61], which turns this system into the only one in the current market that allows a fully automatic measurement of androstenone and skatole. The REIMS technique eliminates the sample excision and sample preparation; the iKnife can be used at the slaughter line due to its cable connection length (4 m) with the body of the device placed in a separate room under appropriate environmental conditions [64]. Also Raman devices can, in principle, be used without sampling or sample preparation. A truly portable version of this technology would allow us to classify boar taint levels at the slaughter line, but still needs to be adapted to work at line speed. The final determination of the content of the boar taint compounds is not direct, but it is determined by the calibration of the Raman device and the later chemometric analysis of the spectra [75].

### 3.4. Speed and Capacity

The **capacity** of a technique is an important characteristic determining the suitability to be used on/at line. If the capacity is high, this is a strength and if it is low this is a weakness as the method is time-consuming. The capacity depends on several time-consuming factors such as the sampling and sample pre-treatment, analysis speed and the time to obtain the results. A very short measurement time with immediate results can be obtained with the human nose or trained sensory assessors on line. However, it has the disadvantage that the assessors may get tired and saturated and, as per industry reports, need to be replaced approximately every 30 min [37,56]. In fact, a prolonged exposure to only one substance/odour causes a reduction in the intensity of sensation, triggered by a reduced excitability of the receptors [99].

REIMS has the advantage that it does not need sample pre-treatment and this favours its speed but it requires a high cleaning frequency (every 10 samples; [38]), thus reducing the effective speed.

Sample pre-treatment in LDTD-MS/MS technology increases the total measurement time, while analysis time is low (8–10 s; [31,60]). Total time needed to obtain the results is reported to be less than 40 min [31]. The full automation of the sampling and sample pre-treatment of LDTD-MS/MS (from DTI) allows work to take place in a continuous way and enables us to process and obtain results of 360 samples/h, i.e., 2880 samples per 8 h working day. Thus, this technology, linked with the automation, allows us to have a high capacity and is quick enough to be suitable for boar taint carcass classification at the abattoir before carcasses reach the cold storage room [31,61].

### 3.5. Performance

Regarding the performance, it is difficult to compare between various measurement principles and/or studies. This difficulty is related mainly to three aspects: the reference sample used as gold standard [26,100], the indicator used to determine performance (i.e., reproducibility, repeatability, sensitivity, specificity, receiver operating characteristics (ROC)-curves) and the way to calculate it (i.e., cut-off criteria, working conditions). Regarding the **gold standard,** this could be (i) the androstenone and skatole levels based on standardized chemical reference methods, (ii) boar taint intensity (or intensities of androstenone and/or skatole odour) or (iii) the consumers’ liking. Consumers’ liking of the pork would probably be the ideal measure since consumers are the last link in the production chain and, consequently, those who will accept or reject the pork if tainted. Nevertheless, there are important differences in consumers’ liking of meat that depend on many different psychological, sensorial and marketing factors [101] (i.e., sensitivity to androstenone and skatole, culinary habits, beliefs, cooking procedure, etc.). Finally, also the sample set used to evaluate calibration and validation may influence the performance.

In **sensory-based methods**, boar taint is usually used as gold standard. One possibility is to use the average panel evaluation when more than one assessor evaluates the same sample [26,100]. The performance of the human nose methodology depends on the olfactory acuity of the assessors and their training and motivation as well as the gold standard used [26,47]. The performance of sensory evaluation can usually be increased if a group of assessors (i.e., more than one) is used [26] and if assessors are re-trained periodically. This could be a threat because it will increase the cost of the evaluation. A balance between the performance and the cost should be considered by the slaughter plants. Every company will have their own strategy which will consider the risk that they are able to assume in relation to sensitivity and specificity (false positive or false negative samples). Sensitivity and specificity were also determined in Raman technology showing values within the range of those reported by the human nose [75], probably because the range is very variable.

In **instrumental methods**, usually the chemical detection of androstenone and skatole is used as a gold standard, but the way to analyse them is very variable, hindering the direct comparison between published results. Since 2014 there has been a new chemical reference method for androstenone and skatole determination in the EU [35,36]; however, it is not the most used method. The best way to compare and validate the method performance, would be to use standardized reference samples for all the technologies in a slaughter house setting. Data show that methods such as LDTD-MS/MS is robust and accurate [61] while other methods like REIMS and Raman spectroscopy are not that accurate [38,75,78] and since they are also calibration dependent, they will probably be less robust when implemented in different conditions.

### 3.6. Investment Costs

The **cost** of the methodology is also an important parameter to take into consideration, but it is difficult to compare because limited information is provided for most of the methodologies. Instrumental methodologies, such as LDTD-MS/MS need an important initial investment, but the final cost per sample is estimated to be lower than 1 € [61] considering only consumables; this likely depends on the depreciation, which likely differs depending on the throughput of a slaughter plant. No information about the cost per sample for the other methodologies is available. Sensory-based methods, such as human nose, need less initial investment, but the personnel costs can be high because several assessors are required to allow alternance between them and, moreover, periodical training is needed. Furthermore, if it is decided that carcasses should be evaluated by more than one assessor to reduce the risk of misclassification, this would also affect the costs. The cost of sample evaluation depends on the salary of the operator, the line speed and the proportion of male carcasses to be measured. The costs of the assessors can be reduced if they can do other tasks at the abattoir, thus combining sensory assessment with other operations, which is mostly the case. For Raman, Biosensors and REIMS methodologies an operator is needed to handle both the sampling, sample preparation and the measurement. Within the BoarCheck project [37], an estimation of the total costs (operational plus investment, without considering reagents and maintenance costs for the instrumental methods) for sensory-based and instrumental-based methods was carried out considering a slaughter plant of 9000 pigs/week (3 lines and 2 shifts/day). It was estimated that on line sensory measurements were 0.1€ lower than on line instrumental mass spectrometry based measurements, while the on line sensory method was 0.63€ more expensive than the at line instrumental one.

### 3.7. Other Considerations

Before choosing a methodology for boar taint classification on/at line, some characteristics of the slaughter plant, such as the line speed, the total number of **slaughtered pigs per day** as well as the **number of entire male carcasses per day** need to be considered. According to the literature, colorimetric and LDTD-MS/MS methods can evaluate around 360 samples/h [37,59,61] and, human nose can work in a line speed up to 650 samples/h [56], although this can differ between assessors. With this information and knowing the amount of carcasses to be evaluated every hour (which depends on the speed line and proportion of entire males), the slaughter plants needs to consider the technical feasibility of these methods according to their needs. In any case, the capacity of the methods can be increased by running in parallel more than one device or assessor, and automate the sampling and sample preparation, if needed. In fact, this solution needs to be considered, because, for instance, for huge slaughter plants with a high proportion of entire males, this would be the only feasible solution.

In addition, the **turnover** of the abattoir is an important factor in determining the economic feasibility of the different solutions. Some technologies are very expensive and only feasible in (very) large abattoirs where many male carcasses are slaughtered. Some other technologies are more affordable and feasible for smaller slaughter plants or slaughter plants with a low proportion of entire male pigs.

Thus, cost-effectiveness, the characteristics of the abattoir together with the performance, availability and possibility of automation of the technology, maintenance service, as well as the commercial importance of having a sorting system, are factors that will steer the industry to determine the most suitable technology that meets their needs and possibilities.

All of the above-mentioned methodologies perform the evaluation or the sampling in one specific place of the carcass, usually the subcutaneous fat of the neck region, and this is another important issue that may affect the final results. Skatole content depends on the place of sampling in the carcass [102,103], although some studies did not report significant intra-carcass variation [104]. Regarding androstenone content, no systematic or significant differences depending on the place of sampling were reported in most of the studies [103,105,106] with some exceptions [107]. Furthermore, systematic variations in the levels of androstenone and skatole through the subcutaneous neck fat cross section have been reported [104]. Standardization and harmonization of the sampling location is advisable when results need to be compared.

Innovation in grading systems that go beyond lean meat and boar taint by including important technological meat quality traits such as intramuscular fat, fatty acids composition and water holding capacity and eating quality traits, would be of great interest for the meat industry. This would allow a better classification of meat, optimize its processing and help to reach the right market according to the product characteristics and the consumer demands. This could be done by using several types of classification devices or using **multi-uses devices** that are able to predict more than one characteristic. LDTD-MS/MS, REIMS and Raman have this potential, which is a great opportunity and benefit of these methods.

## 4. Conclusions

This overview of the different methodologies to determine boar taint at/on line, allows us to conclude that the technology that arises as most promising and readily available is the combination of laser diode thermal desorption and 2 dimensional mass spectrometry (LDTD-MS/MS). It automatizes both the sampling and sample preparation and it allows work to be undertaken at line, is fast and robust and measures both androstenone and skatole with high accuracy and precision. It appears to be a viable option for (very) large slaughter plants where the number of carcasses to be classified is big enough to pay off the technology. In smaller slaughter plants, sensory evaluation is suitable. It is a direct assessment of the sensory perception and, if assessors are well trained, it is robust enough to classify carcasses. However, it should be taken into account that sensory evaluation (human nose scoring), due to its speed, can also be used in large slaughter plants with satisfactory results. Finally, the economical and practical constraints will determine which method is best suited, depending on the share of entire male pigs, the importance of classification based on sensory or chemical analysis for their markets, the ease of implementation, the availability, and the price. Also, it will be a decision of the slaughter plants to use the classification for sorting out carcasses and/or for payment. The other technologies that we evaluated, namely, electrochemical biosensors, REIMS and Raman spectroscopy, still need to be further optimized and validated within abattoir conditions; their performance in these conditions needs to be determined. Compared to the study performed in 2012 within the BoarCheck EU project, improvements in sensory evaluation methodologies have been performed and have been implemented in more slaughter plants. In addition, LDTD-MS/MS has evolved and is entering the market. Other technologies have emerged as potentially applicable at slaughter plant level such as electrochemical biosensors, Raman spectroscopy and REIMS.

## Figures and Tables

**Table 1:** Strength, Weaknesses, Opportunities and Threats (SWOT) analysis of the six compared methods according to readiness (**R**), type of measurement (**T**), traceability and sampling pre-treatment (**S**), Speed and capacity (**C**), performance (**P**), investment costs (**I**), and other aspects (**O**).

	Internal Factors		External Factors	
	Strengths	Weaknesses	Opportunities	Threats
**Human nose**	**R:** Already applied in abattoirs, TRL-9 **T:** Online as well as at line, determine sensory perception of boar taint S: Does not require sampling if on line C: Immediate result P: it can easily identify carcasses with high levels of boar taint I: Low initial investment	**T:** Based on human performance, no quantification of AND and SKA, need of initial and periodic training, level of detection intrinsic to the assessors, need of turn-over of assessors due to fatigue **S:** Needs sampling plan to keep traceability if at line **P:** High variability in accuracy, high training efforts are needed to identify carcasses with low levels of boar taint, results from individual assessors cannot be linked to consumer acceptance in a scientific/objective way	**C:** Adaptable to an increase of productivity (more assessors) **I:** Easy to implement at slaughter line (no big infrastructures needed) **O:** Possibility to give feedback of boar taint content to genetic companies/breeding programs	C/I: High speed of the lines requires more assessors, so higher costs emerge **P/I:** Higher performance goals require multiple assessors **O:** It is a short-term strategy because probably it will be replaced by automatic methods when available.
**Colorimetric method**	**R:** Used in abattoirs for many years, TRL-9 **T:** Quantitative SKA equivalents, objective, direct measurement **C:** Result available after chilling before enter cooling room **P:** Robust	**T:** Only SKA equivalents determination (AND not measured) **S:** At line, need of sampling plan to keep traceability, need of sampling pre-treatment **I:** High initial investment **O:** Environmental contamination (chemical residues)	**I:** Relatively easy to implement at slaughter line (no big infrastructures needed) **O:** Possibility to give feedback of SKA equivalents content to genetic companies/breeding programs	
**LDTD-MS/MS ^1^**	**R:** Already available at the market, TRL-8/9 **T:** Quantitative SKA and AND, objective, direct measurement **C:** Long intervals between maintenance, result available after chilling before entering to the cooling room, 360 samples/h (DTI ^3^) **P:** Robust **I:** Analytical cost 1€/sample (DTI ^3^) (including consumables and excluding personnel, maintenance, depreciation of investment)	**S:** At line, need of sampling plan to keep traceability, need sampling pre-treatment **I:** High initial investment (not only LDTD-MS/MS device) **O:** Environmental contamination (chemical residues), need of disposable items to minimize cleaning and cross-contamination	**C:** Possibility of automation **O:** Possibility to give feedback of AND and SKA content to genetic companies/breeding programs, equipment could be used for determination of other compounds (not related to boar taint) **P:** Relationship between AND/SKA levels and consumer acceptance partly known [87,88] and is currently being further investigated.	**I:** High speed lines would need adjustments of the device, personnel, maintenance and depreciation costs not included in the 1€ per sample. Probably highly variable according to slaughter plants and chains.
**REIMS ^2^**	**T:** Objective **S:** On line does not require sampling or sample pre-treatment. **I:** Low operational cost	**R:** TRL-5 **T:** Indirect measurement, dependent on discriminant models’ training set, classification in yes-no taint **C:** Needs maintenance (cleaning) after low number of samples **I:** High initial investment **O:** Environmental contamination (chemical residues), need of disposable items to minimize cleaning and cross-contamination	**I:** Relatively easy to implement at slaughter line (no big infrastructures needed) **O:** Equipment can be used to determine other compounds (not related to boar taint)	**P:** Variability of carcass characteristics in different slaughter plants (could decrease robustness), actual performance in industrial conditions (relationships of results with AND/SKA levels or consumer acceptance) still unknown. **I:** High speed lines would need adjustments of the device
**Raman spectroscopy**	**T:** Objective **I:** Low analytical cost	**R:** TRL-4 **T:** Indirect measurement of boar taint or AND/SKA, dependent on discriminant models’ training set or calibration set **S:** At line needs sampling plan to keep traceability, need sampling pre-treatment **C:** High acquisition time	**R:** Portable device exists, and it could be implemented on slaughter line provided that measurement/data acquisition become vaster without affecting performance **O:** Equipment can be used to determine other compounds (other than boar taint), possibility to give feedback of boar taint classification (Raman) or AND and SKA content (SERS) to genetic companies/breeding programs	**P:** Variability in slaughter plant carcass characteristics (could decrease robustness), actual performance in industrial conditions (relationships of results with AND/SKA levels (SERS)/boar taint classification (Raman) or consumer acceptance) still unknown **I:** High speed lines would need adjustments of the device
**Electrochemical biosensors**	**T:** Quantitative SKA and AND, objective **S:** Does not require sampling or sample pre-treatment. **C:** Result available after chilling before entering the cooling room	**R:** TRL-6 **S:** Need to keep traceability due to time lapse **I:** Need of disposable items to minimize cleaning and cross-contamination **O:** Environmental contamination (if disposable)	**C:** Possibility of automation **P:** Relationship between AND/SKA levels and consumer acceptance partly known [87,88] and is currently being further investigated **O:** Equipment can be used to determine other compounds (other than boar taint), possibility to give feedback of AND and SKA content to genetic companies/breeding programs	**I:** High speed lines would need adjustments of the device, costs (disposable probes, personnel, maintenance, depreciation) unknown

AND: androstenone; SKA: skatole; TRL: Technology Readiness Level; ^1^ LDTD-MS/MS: Laser Diode Thermal Desorption and mass spectroscopy; ^2^ REIMS: Rapid evaporative ionization mass spectroscopy; ^3^ DTI: Danish Technological Institute solution.
